# *Bibliometric Analysis* of Parasitological Research in Iran and Turkey: A Comparative Study

**Published:** 2013

**Authors:** A Rashidi, B Rahimi, M Delirrad

**Affiliations:** 1 Department of Information Sciences, School of Medicine, Urmia University of Medical Sciences, Urmia, Iran; 2 Department of Medical Informatics, School of Medicine, Urmia University of Medical Sciences, Urmia, Iran; 3 Department of Forensic Medicine and Clinical Toxicology, School of Medicine, Urmia University of Medical Sciences, Urmia, Iran

**Keywords:** Bibliometric indicators, Citation analysis, Iran, Turkey

## Abstract

**Background:**

This study was designed to assess and compare the quantity and quality of Iranian and Turkish researchers working in the field of Parasitology from bibliometric point of view.

**Methods:**

To assess the contributions and achievements of the Iranian and Turkish parasitologists, bibliometric analysis was carried out based on the citation data retrieved from Web of Science.

**Results:**

The absolute productivity of Turkish and Iranian parasitologists’ papers has almost tripled for Turkey, from 12 papers in 2002 to 36 papers in 2011, and decuple for Iran, from 10 papers to 123 from 2002 to 2010. The average number of citation per article is about 5.8 and 4 for Turkish and Iranian parasitologists’ papers, respectively. The “Veterinary Parasitology” journal was the most cited journal in both countries. The majority (more than 90%) of cited items was foreign journal articles and one half of all references in journals articles dated 11 and 12 years while one half of cited books was dated within 14 to16 years for Turkish and Iranian papers, respectively.

**Conclusion:**

Based on observed data and applied model, it is anticipated that the total number of Iranian and Turkish parasitologists’ publications in Web of Science will exceed of 2512 and 240 articles per annum for Iranian and Turkish in 2020, respectively.

## Introduction

Scientific progress is one of the most important indicators for the social and economic development (1). In developing countries, where improvements in healthcare and medicine are most needed, knowledge creation, and especially, applications of findings are key factors in their development (2, 3).

During 2000s both the number of universities and research institutions and academic members has grown considerably In Iran and Turkey. Based on diverse reports, Iran and Turkey, during the past decade had a noticeable contribution in science. For instance, Iran and Turkey had remarkable input on parasitological research, as each country has succeeded to include, at least, one parasitology journal to be indexed in Web of Science (Iranian journal of parasitology and Turkiye parazitoloji dergisi= Acta parasitologica Turcica) (4–6).

From a single Iranian paper indexed in Science Citation Index (SCI) in 1972 (7), in conjunction with the fast growing scientific publications elsewhere in the world, studies of and Osareh and Wilson(8) have shown Iran to have been making considerable movements towards collaboration in the world of scientific productivity. The same is true for Turkey as the first paper related to the field of parasitology was published in 1977. Struggling to improve both countries’ position in the world of science, researchers have been encouraged to publish their findings in highly ranked international scientific journals (9). The main sources for such measurements have been the bibliographical databases compiled by the Institute for Scientific Information (ISI) (3). Citation analysis traces, between scholarly works can assist in the identification of the origin and impact of ideas and thereby the assessment of contribution in the making of scientific knowledge (10).

This research focuses on the bibliometric indicators to identify mainly: a) the format of materials used in Iranian and Turkish parasitological research, b) the age of cited items, c) the most frequently used journal titles which are critical to maintaining a core collection; and d) the half life of the most cited journals.

## Methods

The Web of science database was queried based on the term “Iran and Turkey” in the “address” field and refined by “Parasitology” as a subject category on 31 December of 2011. There were 323 and 678 publications that met the selection criteria for Turkey and Iran, respectively.

The bibliographic data were transferred to Microsoft Excel™. Further confirmation of the author's affiliation was obtained by checking the address for the authors.

The dataset was examined from different perspectives, including year of publication, type of publication, the most productive authors, institutions or universities, the authorship pattern, core subject areas and journals. In addition citation data of the articles published in journals indexed in Web of Science were analyzed separately, from different points of view. The citation half-life for each of the most cited journals’ titles were then calculated by working out the time taken to receive 50% of the total number of citations from the current publication year backwards.

## Results

Until 31th December 2011, 323 and 678 articles were indexed in web of science by Iranian and Turkish researches on Parasitology and its related subject areas.

Table 1 shows the growth rate of parasitological publications from Iran and Turkey in the WoS. The absolute productivity of Turkish and Iranian parasitologists’ papers has almost tripled for Turkey from 12 papers in 2002 to 36 papers in 2011and ten times for Iran from 10 papers to 97 at the same time. Interestingly a more sophisticated analysis revealed that the percentage of growth is in favor of Turkey. The following table and figure have been developed to shed light this aspect.

**Table 1:** Trend of articles published by Iranian and Turkish parasitologists in journals indexed in Web of Science from 1972 to 2011

Publication Year	1972	1973	1974	1975	1976	1977	1978	1979	1980	1981	1983	1984
No of article (Iran)	1	6	4	2	1	3	4	3	5	4	1	1
No of article (Turkey)	0	0	0	0	0	0	0	1	0	0	0	1
Publication Year	1987	1988	1990	1991	1992	1993	1994	1995	1996	1997	1998	1999
No of article (Iran)	0	3	0	0	1	2	2	6	2	1	10	6
No of article (Turkey)	1	1	1	1	0	2	1	1	2	1	3	4
Publication Year	2000	2001	2002	2003	2004	2005	2006	2007	2008	2009	2010	2011
No of article (Iran)	7	7	10	13	22	18	47	63	95	108	123	97
No of article (Turkey)	4	8	12	18	19	25	29	42	41	36	30	36

### Authorship

**Fig. 1:**
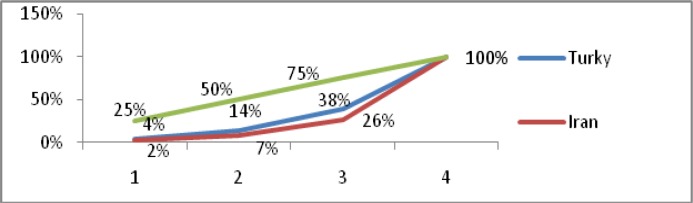
The percentage of authors’ contribution in relation to the percentage of articles production

The last few decades have witnessed a growth in collaborative endeavours as a study (11) demonstrated that, in general, the impacts of UK papers in any discipline or sector are higher if there is a collaboration of some kind. To see authorship pattern within publications indexed in WoS by Iranian and Turkish researchers working on papasitological matters Table 2 and Fig. 1 serve to illustrate the model.

**Table 2:** The collaboration pattern of Iranian and Turkish scientists’ publication indexed in Web of Science

No of authors’ collaboration	% of papers In Turkey	% of papers In Iran
Single author	3.7	6.0
2 Authors	13.9	9.6
3 Authors	19.2	20.4
4 Authors	16.7	18.9
5 Authors	13.0	14.5
6 Authors	14.6	11.9
7 Authors	5.3	6.8
8 Authors	5.3	4.7
9 Authors	2.2	3.4
10 Authors	2.5	1.9
11 Authors	0.9	1.3
12 Authors	0.9	0.3
13 Authors	0.6	0.3
14 Authors	0.6	0.3
>15 Authors	2.8	0.3
Total no of papers	323	678

With respect to the authors’ collaboration, the above table shows that, overall, about 96% in Turkey and In Iran about 94% of papers were written in multiple-author status.

Fig. 1 shows that 2% of Iranian and 4% of Turkish of authors were responsible for the 25% of articles production. Fifty percent of articles had been published by 7% and 14% and 75% of articles were produced by 26% and 38% of authors in Iran and Turkey respectively. The figures implicitly indicate that the responsibility and accountability are more shared among Turkish parasitologists compared to Iranian parasitologists. The collected data allowed for author productivity to be measured on the basis of the number of articles published. The most prolific authors In Iran were Mohebali, M. with 47 and Vatandoost, H. with 34 papers and in Turkey the most productive authors were Aktas, M. with 18 and Dumanli, N. with 13 papers, respectively.

### Most cited articles in WoS

The objective of the following analysis is to identify and list the articles that have influenced others the most as measured by citation count. An understanding of which research is viewed by the research community as most valuable to build upon may provide valuable insights into what research or even researcher to focus on now and in the future (12). Citation data being available for articles indexed in WoS shows that 323 and 678 published articles by Turkish and Iranian parasitologists received 1884 and 2726 citations. In other words, the average number of citation per article is 5.8 and 4 for Turkish and Iranian papers, respectively. Among them 40% of Iranian and 27% of Turkish articles had not received any citations by the time of the analysis. Based on the analysis, a list of the 3 most cited articles is presented in Table 3.

**Table 3:** The 3 most cited articles of Turkish and Iranian parasitologists in WoS

Iranian papers				Turkish papers			
Authors	Title	Time cited	Year of publication	Authors	Title	Time cited	Year of publication
Ok, et al.	Leishmaniasis in Turkey	49	2002	Mohebali et al.	Epidemiological aspects of canine visceral leishmaniasis in the Islamic Republic of Iran	55	2005
Altintas	Past to present: echinococcosis in Turkey	48	2003	Dalimi, A and et al.	Echinococcosis/hydatidosis in western Iran	54	2002
Hurst, et al.	Adoniavariegata (Coleoptera: Coccinellidae) bears maternally inherited Flavobacteria that kill males only	47	1999	Hashemifesharki, R	Control of theileria-annulata in Iran	41	1988

### Journal Titles

The three top ranking journal titles in which Iranian and Turkish parasitologists published their papers for Turkish papers are “VET PARASITOL”, “PARASITOL RES” and “J CLIN MICROBIOL” with 202,180 and 100 indexed papers respectively. With regard to the Iranian papers, the distributions of articles’ journal titles are “VET PARASITOL”, “AM J TROP MED HYG” and “PARASITOL RES” each with 688, 483 and 360 published papers.

In preferred journal titles of parasitologists of both countries, “Vet Parasitol” was the first dominant journals.

### Affiliations

The addresses of all authors were assessed to determine most productive Iranian- and Turkish based universities or institutions. The results of top-ranked Iranian and Turkish researcher's affiliations publications in journals covered by WoS for Turkey are “Firat Univ”, “Ondokuz Mayis Univ”and “Ege Univ” each with 28,17and 16 papers and the Iranian top universities were “Univ Tehran Med Sci”, “Inst Pasteur” and “Univ of Tehran” each with 134, 65 and 59 published papers, respectively.

### Topics

The distribution of subject categories of Iranian and Turkish parasitologists’ articles indexed in WoS shows that in both countries around 64% of articles were directly related to the parasitology. Further analysis of counts of Iranian and Turkish parasitology papers’ cross pollination in different specialties reveals that the cross pollination difference within two subject categories is significant. While for the veterinary subject area Iranian parasitologists had not paid attention, Turkish parasitologists wrote about 12% of their papers on that category. The vice versa is nearly true for public environmental and occupational health.

### Type of Articles

An analysis of the types of published papers in WoS was also carried out. The results are summarized in Table 4. Accordingly more than 92% of the papers were original research articles. Of these, around 4% are proceedings paper, the rest being either letters or editorial materials or reviews.

**Table 4:** Types of published papers by Iranian and Turkish parasitologists

Type of articles	Frequency for Turkey	Percentage	Frequency for Iran	Percentage
Article	301	93.2	627	92.1
Proceedings paper	12	3.7	30	4.4
Review	11	3.4	4	0.6
Letter	3	0.9	5	0.7
Editorial material	2	0.6	1	0.1
Note	2	0.6	11	1.6
Correction	1	0.3	1	0.1
Meeting abstract	0	0.0	2	0.3

### The internationally published leading parasitological journals

To reveal to what extent Turkish and Iranian parasitologists’ cited journals coincide with the internationally accepted prototype; the most cited foreign journals and their corresponding percentiles are presented in Table 5. These might be served to categorize the three steps in weeding and archiving decisions. This threshold can be adjusted to meet the needs of individual universities, depending upon factors such as available space.

**Table 5:** List of 15 core journals related to parasitological subject matters ranked by total cites impact, 5-year impact and impact factor (IF) in descending order

Rank	Abbreviated Journal Title	Total Cites IF	IF 5-Year	IF
1	Plos Pathog	10833	9.079	9.675
2	Trends Parasitol	4464	4.906	5.285
3	Plos Neglect Trop D	2020	4.752	4.849
4	Int J Parasitol	8331	3.822	3.938
5	Malaria J	4012	3.489	3.551
6	Mol Biochem Parasit	7649	2.875	2.963
7	Parasitology	7396	2.522	2.53
8	Parasite Immunol	2420	2.357	2.299
9	Vet Parasitol	9727	2.331	2.458
10	Acta Trop	4527	2.262	2.5
11	Parasitol Int	1190	2.259	2.366
12	Parasite Vector	272	2.13	2.14
13	Mem I Oswaldo Cruz	5385	2.058	2.081
14	Exp Parasitol	4218	1.869	1.841
15	Parasitol Res	5741	1.812	1.723

Over the course of the years under investigations, 16980 citations representing 100% of the total number of references of articles indexed in WoS for Iran and 2962 for Turkey have been analyzed and journals were grouped according to Bradford's Law of scattering (13) to determine zone 1 which consists of a few journals and have received the largest number of citations.

In Tables 6–7, the top most cited journals are ranked in descending order. The table also lists first, second and third quartiles of usage for each title. These journals were able to provide more than 28% and 33% of information needs of Iranian and Turkish parasitologists respectively. These can be used for classification of the three chronological steps in weeding and archiving decisions. Again this threshold can be adjusted to meet the needs of individual universities, depending upon factors such as available space.

**Table 6:** The 15 most cited journals by Iranian parasitologists and their corresponding quartiles in 2011

*Rank	Time cited	Journal	25	50	75	In Shelf	Active Archive	Passive Archive
**1**	695	Vet Parasitol	2005	2003	1998	6	8	13
**2**	492	Am J Trop Med Hyg	2003	1998	1990	8	13	21
**3**	395	T Roy Soc Trop Med H	2002	1994	1983	9	17	28
**4**	361	Parasitol Res	2007	2005	2000	4	6	11
**5**	316	Int J Parasitol	2004	2000	1995	7	11	16
**6**	312	J Parasitol	2000	1993	1972	11	18	39
**7**	308	Ann Trop Med Parasit	2003	1997	1986	8	14	25
**8**	296	Parasitology	2003	1999	1992	8	12	19
**9**	286	Mol Biochem Parasit	2001	1995	1992	10	16	19
**10**	276	J Clin Microbiol	2003	2000	1995	8	11	16
**11**	274	Infect Immun	2003	1998	1994	8	13	17
**12**	241	Acta Trop	2006	2003	1997	5	8	14
**13**	217	Iran J Public Health	2006	2003	1996	5	8	15
**14**	204	Exp Parasitol	2007	2000	1990	4	11	21
**15**	197	Vaccine	2006	2004	2001	5	7	10

* Ranked by number of citations

**Table 7:** The 15 most cited journals by Turkish parasitologists and their corresponding quartiles in 2011

*Rank	Time cited	Journal	25	50	75	In Shelf	Active Archive	Passive Archive
**1**	203	Vet Parasitol	2007	2004	1999	4	7	12
**2**	180	Parasitol Res	2008	2007	2004	3	4	7
**3**	100	J Clin Microbiol	2004	2000	1995	7	11	16
**4**	79	Int J Parasitol	2005	2002	1998	6	9	13
**5**	69	Parasitology	2007	2003	1999	4	8	12
**6**	55	Am J Trop Med Hyg	2004	2002	1997	7	9	14
**7**	55	Acta Parasitol	2002	1996	1998	9	15	13
**8**	45	Acta Trop	2008	2004	2002	3	7	9
**9**	34	J Parasitol	2003	2000	1998	8	11	13
**10**	31	Comp Parasitol	2006	2006	2005	5	5	6
**11**	30	Mol Biochem Parasit	1998	1993	1992	13	18	19
**12**	29	Trends Parasitol	2007	2004	2002	4	7	9
**13**	26	Res Vet Sci	2007	2005	1986	4	6	25
**14**	25	Ann Trop Med Parasit	2004	2001	1990	7	10	21
**15**	25	Parasitol Int	2008	2006	2005	3	5	6

* Ranked by number of citations.

The “VET PARASITOL” Journal was the most cited journal in both countries. The second most-used journal was “AM J TROP MED HYG” with 429 by Iranian and “PARASITOL RES” with 180 times of citation by Turkish parasitologists. The journal of “T ROY SOC TROP MED H” took third place with 395 by Iranian and “J CLIN MICROBIOL” with 100 times of citation by Turkish parasitologists.

As may be expected, the specialized journals are ranked the highest, whereas journals covering a broad range of parasitological subjects such as Vaccine tend to be cited less frequently.

One of the recognitions on the importance of a journal by the international community is the inclusion of the journal in the prestigious databases. The “IRAN J PUBLIC HEALTH” being indexed in ISI database was among frequently cited journals by the Iranian parasitologists with 217 times of citation. It seems that the half-life of “PARASITOL RES” is relatively lower in both countries publications.

### Usage of information resources by Iranian and Turkish parasitologists

To investigate the types of information sources used by Iranian and Turkish parasitologists and their preferred information formats several queries were written to extract relevant information. Table 8 shows the number and percentage of each type of information sources, which were cited by Iranian and Turkish parasitologists for the articles indexed in WoS. Table 8 shows that about 90 percent of the total citations were to Journals, 8-9 percent to Books. There was no citation to web resources.

**Table 8:** Different information sources usage over the time for the articles indexed in Web of science from 1972 to 2011

	Turkey			Iran		

Type of Media	N	Percent	Half life	N	Percent	Half life
Journal	2963	89.2	11	16879	90.5	12
Book	322	9.7	14	1427	7.6	16
Thesis	18	0.5		131	0.7	
Conference material	12	0.4		139	0.7	
Report	5	0.2		36	0.2	

### Trends in the number of articles indexed in WoS journals since 2001

The data for parasitological research based on papers indexed in WoS were analyzed in the present study (Table) to see whether or not the trend found could be extrapolated to predict later growth. Based on distribution of observed articles published between 2001 and 2010, the relationship between the number of articles indexed in WoS by Iranian and Turkish parasitologists (X) and the year of publication (Y) was found to fit an exponential model with the following formula for Iran and Turkey as follows:

Iran *Y* = 3 .987 *EXP* ^0.326x^

Turkey *Y* = 9 .658 *EXP* ^0.159x^

The model has been used to construct Table 9 showing the expected number of Iranian and Turkish article publications over the next few years.

**Table 9:** Average number of published medical articles, based on SCI searches

Turkey				Iran			

Year	Observed (per annum)	Year	Predicted from Model (per annum)	Year	Observed (per annum)	Year	Predicted from Model (per annum)
2000	4	2011	57	2000	7	2011	138
2001	8	2011	57	2001	7	2011	138
2002	12	2012	67	2002	10	2012	190
2003	18	2013	78	2003	13	2013	263
2004	19	2014	92	2004	22	2014	363
2005	25	2015	108	2005	18	2015	501
2006	29	2016	126	2006	47	2016	692
2007	42	2017	148	2007	63	2017	955
2008	41	2018	174	2008	95	2018	955
2009	36	2019	204	2009	108	2019	1820
2010	30	2020	240	2010	123	2020	2512

Based on observed data and applied model, it is anticipated that the total number of Iranian and Turkish parasitologists publications in WoS will exceed of 2512and 240 articles per annum in 2020, respectively.

## Discussion

The parasitological research output analyzed in this study demonstrated a clear pattern of disseminating research to their readers. This dissemination happened in two ways. First, more than 90% of all the papers published in the time period of our study were original articles. Second, in addition to articles that presented research studies on parasitology, about 90% of references used by authors to develop publications of all types were articles followed by proceeding papers (around 8%). In the other word, when parasitological references were cited, most of these (90%) were to research articles than books, similar to other biomedical and hard sciences (14–16), suggesting the importance of parasitological research in the development of parasitology knowledge. In the medical basic science, Larivière (15) and colleagues found that 93% of all references were citations to journal articles.

Because many parasitologists uses journals as part of their professional materials, having access to their favorite journals, for example, in their departments may encourage them to read about research relevant to their practice updates.

According to Estabrooks, a cross pollination referencing pattern such as parasitology and veterinary indicates a field that is “not closed or insular…(but) is open to the infusion of knowledge from other disciplines”^10^.

The mean number of citations was 20 per article. Price (17) reported that research articles in hard sciences had an average of 22 references, which was used as a benchmark in a citation analysis study by Vincent and Ross (16). MacRoberts and MacRoberts (18) reported that the average number of citations in biomedical articles was about 20. The citation rate in our study was consistent with those studies. The average number of authors per article was about 5 persons in both countries, comparable with the mode number of authors per article in clinical and life science in Croatia (19).

Although, in both countries, about 96% of Turkish and 94% of Iranian papers have been written in multiple-author status and therefore the trend is toward multi-authorship, but the majority of items published by Iranian researchers have two, three, four, five and six authors. The average number of authors per item was about 4.6 in Iran and 5 in Turkey.

Based on the author's pervious research, making some incentives such as supporting authors for the papers accepted to be presented in foreign conferences and seminars significantly affected the rate of publication in internationally published journals (20). Therefore it can be seen that the proceedings papers took the second place among cited materials. It is very likely that the growth of original articles is partially due to the growth of number of proceedings paper.

Disseminating research in parasitological journals and using research knowledge as a basis for developing those publications may increase parasitologists’ awareness of research useful to their practice.

Additional efforts are needed in parasitologists education programs at all levels to prepare them for reading, understanding, and evaluating research findings for use in practice.

## Conclusion

Given that having publications in international journals acquire higher visibility than those in domestic ones (21), Iranian and Turkish parasitologists attempt to increase their publication rate in journals indexed in well-known databases.

Further analysis of papers with regard to the researchers’ collaboration with other countries’ researchers shows that for Turkey, the Japan with 5 co-authored and Australia with 4 co-authored and for Iran Sweden with 5 co-authored and USA with 5 co-authored papers rank first and second, respectively.

Based on the applied model, it is expected that the total number of Iranian and Turkish parasitologists’ paper in WoS to exponentially increase in the near future.

## References

[1] Diener E, Suh E (1997). Measuring quality of life: Economic, social, and subjective indicators. Soc Indic Res..

[2] The PLoS Medicine Editors (2006). The Impact Factor Game. PLoS Med.

[3] Wade N (1975). Citation analysis: A new tool for science administrators. Science..

[4] SCImago (2007). SJR — SCImago Journal & Country Rank. http://www.scimagojr.com.

[5] http://www.info.sciverse.com/scopus/scopus-in-detail/facts.

[6] http://thomsonreuters.com/products_services/science/science_products/a-z/journal_citation_reports/.

[7] Beiki O, Beiki D (2005). Parsmedline: Establishment of a web-based bibliographic database related to iranian health and medical research. J Med Libr Assoc..

[8] Osareh F, Wilson CS (2002). Collaboration in iranian scientific publications. Libri..

[9] Habibi G, Rashidi A, Feldman MD (2006). Emerging concerns about iran's scientific and medical future. Lancet..

[10] Estabrooks CA, Winther C, Derksen L (2004). Mapping the field: A bibliometric analysis of the research utilization literature in nursing. Nurs Res..

[11] Leimu R, Koricheva J (2005). Does scientific collaboration increase the impact of ecological articles?. Bioscience..

[12] Andrews JE (2003). An author co-citation analysis of medical informatics. J Med Libr Assoc..

[13] Nash-Stewart CE, Kruesi LM, Del Mar CB (2012). Does bradford's law of scattering predict the size of the literature in cochrane reviews?. J Med Libr Assoc..

[14] Holsapple CW, Johnson LE, Manakyan H, Tanner J (1993). A citation analysis of business computing research journals. Inform Manage..

[15] Lariviere V, Archambault E, Gingras Y, Vignola-Gagne E (2006). The place of serials in referencing practices: Comparing natural sciences and engineering with social sciences and humanities. J Am Soc Inf Sci Tec..

[16] Vincent A, Ross D (2000). Citation analysis of the decision science journal. Decision Line..

[17] Crawford S (1984). Derek john de solla price (1922-1983). The man and the contribution. Bull Med Libr Assoc..

[18] MacRoberts MH, MacRoberts BR (1989). Problems of citation analysis: A critical review. JASIST..

[19] Puljak L, Vukojevic K, Lovric Kojundzic S, Sapunar D (2008). Assessing clinical and life sciences performance of research institutions in split, croatia, 2000-2006. Croat Med J..

[20] Larsen PO, von Ins M (2010). The rate of growth in scientific publication and the decline in coverage provided by science citation index. Scientometrics..

[21] Zhou P, Leydesdorff L (2007). A comparison between the China scientific and technical papers and citations database and the science citation index in terms of journal hierarchies and interjournal citation relations. J Am Soc Inf Sci Tec..

